# Identification of Social-Media Platform of Videos through the Use of Shared Features

**DOI:** 10.3390/jimaging7080140

**Published:** 2021-08-08

**Authors:** Luca Maiano, Irene Amerini, Lorenzo Ricciardi Celsi, Aris Anagnostopoulos

**Affiliations:** 1 Department of Computer, Control and Management Engineering, Sapienza University of Rome, via Ariosto, 25, 00185 Rome, Italy; amerini@diag.uniroma1.it (I.A.); aris@diag.uniroma1.it (A.A.); 2 Elis Innovation Hub, via Sandro Sandri 81, 00159 Rome, Italy; l.ricciardicelsi@ELIS.ORG

**Keywords:** media forensics, social media platform identification, video forensics

## Abstract

Videos have become a powerful tool for spreading illegal content such as military propaganda, revenge porn, or bullying through social networks. To counter these illegal activities, it has become essential to try new methods to verify the origin of videos from these platforms. However, collecting datasets large enough to train neural networks for this task has become difficult because of the privacy regulations that have been enacted in recent years. To mitigate this limitation, in this work we propose two different solutions based on transfer learning and multitask learning to determine whether a video has been uploaded from or downloaded to a specific social platform through the use of shared features with images trained on the same task. By transferring features from the shallowest to the deepest levels of the network from the image task to videos, we measure the amount of information shared between these two tasks. Then, we introduce a model based on multitask learning, which learns from both tasks simultaneously. The promising experimental results show, in particular, the effectiveness of the multitask approach. According to our knowledge, this is the first work that addresses the problem of social media platform identification of videos through the use of shared features.

## 1. Introduction

**Figure 1:**
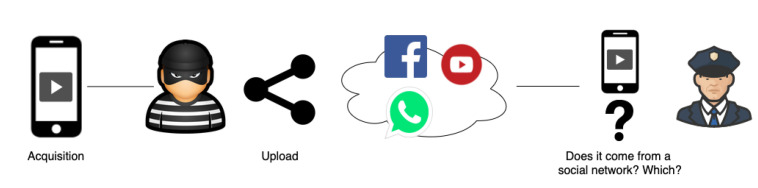
An application example of the proposed solution. An attacker records a video with illegal content and shares it on social networks or messaging apps. Subsequently, the police seize a device with this video and want to trace the source.

Researchers have been studying multimedia forensics for more than two decades in different experimental settings; however, the practical application of these techniques has been limited because of the high variability of real cases, which is difficult to reproduce in experiments. Today, the assessment of the authenticity and the source of multimedia content has become an essential element for building trust in images and videos shared across online platforms. When videos of military propaganda, revenge porn, cyberbullying, or other illegal content are shared on social media, they can easily go viral. While it is important to immediately identify and delete this content from social platforms, another problem to be addressed is to identify the authors of the video to proceed with any legal action. In many other cases, law enforcement may locate a device containing illegal content and to identify its source, it may be necessary to understand whether the video was recorded with the hijacked device or whether it was downloaded via messaging apps or social networks. In fact, in all these cases videos and images could be used as evidence in court and knowing how to identify videos shared on social platforms could help identify criminal networks operating online. However, for this to be possible, it is necessary to be able to prove the origin of such content. In particular, two problems must be solved: (1) Knowing how to reconstruct the source of acquisition (camera model or device) and (2) understanding whether some media content found on an offending device comes from social media. Being able to respond to the latter would allow the sharing network to be reconstructed and possible online criminal groups to be identified. Figure 1 summarizes these two problems.

Deep learning has pushed the design of new methods that can learn forensic fingerprints automatically from data [1,2,3], helping us to take a new step towards applying these techniques to real problems. Despite the promising results of artificial neural networks, some limitations still remain. Single-task learning has been very successful in computer vision applications, with many models performing as well or even exceeding human performance for a large number of tasks; however, they are extremely data dependent and poorly adaptable to new contexts. Recently, collecting data from social networks has become increasingly difficult because of data protection regulations and the most stringent policies introduced by the platforms (https://www.facebook.com/apps/site_scraping_tos_terms.php, https://twitter.com/en/tos—accessed on 4 August 2021). Indeed, it is mandatory to obtain end user consent or the platform’s written permission before acquiring data via the API or web scraping of the most common social networks like Facebook, Instagram or Twitter. Moreover, new data protection regulations, such as GDPR (https://europa.eu/youreurope/citizens/consumers/internet-telecoms/data-protection-online-privacy/index_en.htm—accessed on 4 August 2021), CCPA (https://oag.ca.gov/privacy/ccpa—accessed on 4 August 2021), or the Australian privacy act are contributing to the introduction of new limitations in some countries around the world. All these limitations make difficult to collect enough data to train a deep-learning model. Moreover, the human ability to learn from experience and reuse what has been learned in new contexts is still difficult to reproduce in machine learning as well as in multimedia forensics. All these reasons, along with the unavailability of large training datasets containing both video and image content, have led researchers to treat the problems of social-media–platform identification of images [4,5,6,7] and videos [8] separately. Recently, Iuliani et al. [9] showed that it is possible to identify the source of a digital video by exploiting a reference sensor pattern noise generated by still images taken by the same device, suggesting that images and videos share some forensic traces. Based on this intuition, we build a model that classifies videos from different social-media platforms or messaging apps by taking advantage of the shared features between images and videos. More specifically, to overcome the aforementioned limitations, we try to answer the following question: *Is it possible to decide whether a video has been downloaded from a specific social-media platform? If so, do images and videos have any common forensic trace that can be used to solve video social-media platform identification using both media?* To answer these questions, we propose two methods: A method based on transfer learning and a one based on multitask learning. Both methods offer the possibility of reusing the features learned from one media into another using fewer training data, a feature that is very useful in this domain given the difficulty of finding datasets large enough to train neural networks.

In *transfer learning*, we first train the base model on the image task, and then reuse the learned features, or *transfer* them, to videos. This process tends to work if the features are general, that is, suitable to both tasks [10]. The forensics community has adopted widely transfer learning because, as new manipulation methods are continually introduced, there is a need of detection techniques that are able to detect fakes with little to no training data [11,12]. In *multitask learning*, a model shares weights across multiple tasks and makes multiple inferences in a forward pass. This method has proved to be more scalable and robust compared to single-task models, allowing for successful applications in several scenarios outside the forensic community [13]. Some applications of multitask learning have been even applied to multimedia forensics problems as well, for example, to solve camera model and manipulation detection tasks [14], as well as brand, model, and device-level identification, using original and manipulated images [15].

We apply both learning approaches in this work to accelerate the training of a deep-learning method for deciding whether a video has been downloaded from a social media platform. Because the collection of large datasets for this task is usually very difficult, if not impossible, in practical applications because of privacy reasons, it is worth investigating the effectiveness and the limits of transfer learning and multitasking learning on the task of social media platform identification of videos.

In this paper, we show how well low-level features generalize between images and videos, demonstrating that common platform-dependent features can be learned when the training data are not large enough to train a deep learning model from scratch to estimate the traces left by social media platforms during the upload phase on videos. The sharing process can combine multiple operations that leave different traces in the video signal. These alterations can be exposed in various ways. For example, as first observed in [16], compression and resizing are usually applied by Facebook to reduce the size of uploaded images and this may happen differently on different platforms based on the resolution and size of the input data before loading. As is widely known in multimedia forensics, such operations can be detected and characterized by analyzing the video signal where distinctive patterns can be exhibited. Indeed, these operations typically distort the original video signal with some artifacts that can be detected. When the signal is used as a source of information for the provenance analysis, different choices can be made to preprocess the signal and extract an effective feature representation. After the feature representation is extracted, different kinds of machine-learning or deep-learning classifiers can then be trained to perform platform identification (see Section 3.1). To detect videos shared through social media platforms, we propose two methods that can learn to detect the traces left by different social-media platforms without any preprocessing operation on the input frames. To our knowledge, this is the first work that analyzes the similarity of the traces left by social media platforms on images and videos used in combination. Next, we show that the features learned in the task of social-media identification of images can be successfully applied on social-media identification of videos, but not vice versa, thus suggesting a *task asymmetry*, which could possibly be explained by looking at social-media identification of videos as a special case of the image task. Indeed, as discussed in Section 3.1, shared videos may have both static and temporal artifacts, whereas shared images have static features only. These findings are particularly valuable in investigative scenarios where law-enforcement agencies have to trace the origin of multimedia content without being able to refer to other sources such as metadata. This scenario is depicted in Figure 1.

The rest of this paper is organized as follows: First, in Section 2 we present some related work. In Section 3 we describe our methods and provide a detailed explanation of methods based on transfer learning and multitask learning. In Section 4 we show the experimental results on the VISION dataset [17]. Finally, in Section 6 we draw the conclusions of our work.

## 2. Related Work

When shared on social media platforms and messaging apps, multimedia content is typically subjected to a series of processing and recompression operations to speed up the loading and optimize the display of images and videos on the platform. Videos are typically compressed as sequence of *groups of pictures* (GOP), each of which is made by an alternation of three different kinds of frames: *I-frames*, which are not derived from any other frame and are independently encoded using a process similar to JPEG compression, and *P-frames* and *B-frames*, which are predictively encoded using motion estimation and compensation. While the algorithms used by social platforms are not known, all of these operations leave traces that can be detected [4,6,7,18,19] and, since they typically differ between different platforms [19,20,21], they can be used to distinguish between distinct social networks. According to the survey by Pasquini et al. [21], we can identify two main possible steps in the digital life of a media object shared online, namely the acquisition and the upload. Initially, a real scene is captured through an acquisition device, then, a number of post-processing operations such as resizing, filtering, compressions, cropping, semantic manipulations may be applied. Finally, through the upload phase, the object is shared through social media.

Following these two steps, in the remainder of this section, we describe the state-of-the-art methods that can be used to analyze the acquisition source or integrity of a video (Section 2.1) and to reconstruct information on the sharing history of a video (see Section 2.2).

### 2.1. Forensic Analysis

The main problems in traditional media forensics are the identification of the source of images and videos and the verification of their integrity.

Source-camera identification is the problem of tracing back the origin of a video by identifying the device or model that captured a particular media file. This problem has been very often treated in a *closed-set* setting, meaning that all the devices that we want to be associated with a source video are known in advance. These methods typically rely on Photo Response Non-Uniformity (PRNU) [22]. Houten and Geradts [23] propose video camera source identification of YouTube videos showing the limitations to reach a correct identification on the shared video because of the numerous variations that affect PRNU (e.g., compression, codec, video resolution, and changes in the aspect ratio). Similarly, another work [24] performs an analysis on stabilized and non-stabilized videos proposing to use the spatial domain averaged frames for fingerprint extraction. A different method for PRNU fingerprint estimation [25] takes into account the effects of video compression on the PRNU noise selecting blocks of frames having at least one non-null discrete cosine transform (DCT) coefficient. Other works use PRNU to link social media profiles containing images and videos captured by the same sensor [9,26]. Similar approaches have been introduced for camera model identification [27,28]. Recently, some works have begun to deal with the problem of identifying the source of a video with *limited knowledge* or even an *open-set* of devices. Cozzolino et al. [29] introduce a siamese method based on [2] to estimate camera-based fingerprints (called *Noiseprints*) for video with no need of prior knowledge on the specific manipulation or any form of fine-tuning. Another work [30] from the same research group combines the PRNU and Noiseprint to boost the performance of PRNU-based analyses based on only a few images. In some works [8,31,32] video file containers have been considered for the source identification of videos without a prior training phase. To do this, López et al. [32] introduces a hierarchical clustering method whereas [8] proposes a likelihood-ratio framework. Mayer et al. [33] propose a similarity network based on [1] to extract features from video patches, and to fuse multiple comparisons to produce a video-level verification decision.

Even though most of the techniques described so far are based on deep learning, which has proved successful for camera model identification problems [34], there are other works using different techniques. Marra et al. [35] study a class of blind features based on the analysis of the image residuals of all color bands, where no hypothesis is made on the origin of camera-specific marks, and the identification task is regarded simply as a texture classification problem. Chen and Stamm [36] introduce a model of a camera’s de-mosaicing algorithm by grouping together a set of submodels. Each submodel is a nonparametric model designed to capture partial information of the de-mosaicing algorithm. the diversity among these submodels, leads to the composition of a comprehensive representation of a camera’s de-mosaicing algorithm. Finally, an ensemble classifier is trained on the information gathered by each sub model to identify the model of an image’s source camera.

The application of forgery detection methods on shared videos has been very limited to date. Iuliani et al. [8] show that the dissimilarity between a query video and a reference file container can be estimated to detect video forgery. Mayer and Stamm [1,37] propose a graph-based representation of an image, named Forensic Similarity Graph, to detect manipulated digital images. A forgery can be detected as a separate cluster of patches with respect to the pristine-patches cluster in the graph. Even if the same idea has been applied [33] for video source identification, the robustness of this method has not been tested on forged videos as well.

The next section presents the methods that can be used for the second phase of the pipeline, which is the association of the platform of origin of a video.

### 2.2. Platform Provenance Analysis

Social-media–platform identification has been broadly explored for images. Amerini et al. [7] propose a CNN architecture that analyzes the histograms of image DCT coefficients to reconstruct the origin of images shared across Facebook, Flickr, Google+, Instagram, Telegram and Twitter. Another work [4] introduced a CNN-based model that was used to fuse the information extracted from the histograms of image DCT coefficients with a noise residual extracted from the image content through high-pass filtering. Moreover, by combining DCT features with metadata, Phan et al. [6] showed that is possible to track multiple sharing on social networks by extracting the traces left by each social network within the image file. Finally, PRNU)can be applied as suggested by Caldelli et al. [18] to train a CNN to detect the social network of origin of an image.

The proposal of social-media–platform identification techniques has been instead quite limited for videos. Amerini et al. [38] introduce a preliminary work in which they evaluate different methods to build a fingerprint to detect video shared in social networks and also introduce a method that generates a composite fingerprint by resorting to the use of PRNU noise. Two recent works [8,31] introduced simple yet effective container-based methods to identify video manipulation fingerprints and reconstruct the operating system of the source device, proving the robustness of the method on manipulation introduced by social media platforms. Amerini et al. [39] propose a two-stream neural network that analyze I-frames and P-frames in parallel. All frames are preprocessed converting them from RGB to YUV, and the Y-channel of each frame is used as input for the network. For P-frames, the authors subtract the Gaussian filtered version of the frame from the Y-channel to reduce the noise in these type of frames.

Nevertheless, because these preprocessing operations can change over time, it may be necessary to periodically learn new forensic traces for smaller training datasets. For this reason, in the next section, we propose two learning techniques to train models on little data, possibly taking advantage of what is learned on similar tasks to improve performance and speed up the learning.

## 3. Proposed Method

In this section, we propose a theoretical analysis of what could be the traces that can be left on videos by social media and we propose a framework for platform identification.

### 3.1. The Rationale

**Figure 2:**
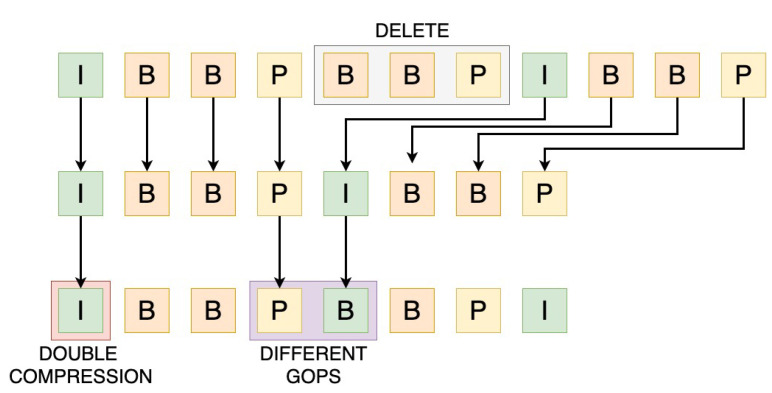
The top line shows an original MPEG encoded sequence. The next lines show the effect of deleting the three frames in the shaded area. The second line shows the reordered frames and the third line the recoded frames. The I-frame before erasing is subjected to double compression. Some of the frames following the deletion move from one GOP sequence to another. This double MPEG compression gives rise to specific statistical and temporal models that can be used to identify the platform of origin.

As discussed earlier, when we upload a video to a social-media platform, it usually goes through a series of operations, which most commonly may include recompression to reduce the bandwidth requirement for using the video on the platform, a resize, and in some cases the removal of some frames of the video to make it fit the maximum duration of the videos imposed by some platforms. While, as mentioned, these operations may vary depending on the platform, in this section we want to formalize as much as possible how these operations can leave information in the video. As shown in [40,41], these operations can leave both static and temporal artifacts in the video signal when a video sequence is subjected to double MPEG compression. Statically, the I-frames of an MPEG sequence are subjected to double JPEG compression. Temporally, frames that move from one GOP to another, as a result of frame deletion, give rise to a relatively larger motion estimation errors. Figure 2 shows an example of a short eleven-frame MPEG sequence. In this example, during the upload phase, the video is subjected to the removal of three frames and subsequent recompression. The second row shows the reordered frames, and the third line shows the re-encoded frames after recompressing the video as an MPEG video.

Statically, when an I-frame gets recompressed with different bit rates (i.e., quantization amounts), the DCT coefficients are subject to two quantization levels, leaving behind a specific statistical signature in the distribution of DCT coefficients [40,42]. Quantization is a pointwise operation, which can be calculated as:

$$Q_{k}\left(s_{1}\right)=⎣\frac{k}{s_{1}}⎦,$$

where $s_{1}$ indicates the quantization step and *k* denotes a value in the range of the input frame. Similarly, double quantization is also a pointwise operation given by:

$$Q_{s_{1}s_{2}}\left(k\right)=⎣⎣\frac{k}{s_{1}}⎦\frac{s_{1}}{s_{2}}⎦,$$

where $s_{1}$ and $s_{2}$ are the quantization steps. From the equation above, double quantization can be described as a sequence of three operations: A quantization with step $s_{1}$, a de-quantization with step $s_{1}$, and a quantization with step $s_{2}$. As Wang and Farid show [41], the re-quantization introduces periodicity of the artifacts into the histograms of quantized frames. As these artifacts will differ depending on the quantization step used by every platform, they can be used to distinguish differences between social media platforms.

Temporarily, deleting a few frames of the video to fit the maximum length set by some platforms can in turn leave information. For example, consider deleting three frames in Figure 2. Within the first GOP of this sequence, the I-frame and the first P-frame come from the first GOP of the original sequence. The third B-frame, however, is the I-frame of the second GOP of the original sequence, and the second I-frame is the first P-frame of the second GOP of the original video. When this new sequence gets re-encoded, we will observe a larger motion error between the first and second P-frames, as they originated from different GOPs. Furthermore, this increase in motion error will be periodic, occurring in each of the GOPs after the frame gets deleted. Formally, consider a six-frame sequence that is encoded as $I_{1},P_{2},P_{3},P_{4},P_{5},I_{6}$. Because of JPEG compression and motion error, each frame can be modeled by an additive noise, that is:

$$I_{i}=F_{i}+N_{i}\;P_{j}=F_{j}+N_{j}$$

with i≠j, where each $N_{i},N_{j}$ is the additional noise and $F_{i},F_{j}$ are the original frames. Notice that the noise for $I_{1}$ through $P_{5}$ will be correlated to each other because they belong to the same GOP, but not to that of $I_{6}$. If we denote the motion compensation as M(·), we can derive the motion error for a frame i,(i>1) as:

$$\begin{array}{cc} e_{i} & =P_{i}-M\left(I_{i-1}\right)\\ & =F_{i}+N_{i}-M\left(F_{i-1}+N_{i-1}\right)\\ & =\left(F_{i}-M\left(F_{i-1}\right)\right)+\left(N_{i}-M\left(N_{i-1}\right)\right). \end{array}$$

Suppose now that we delete frame $P_{4}$, bringing frames $P_{5}$ and $I_{6}$ to the fourth and fifth positions, respectively. $I_{6}$ will now be encoded as the new $P_{5}^{\prime }$. The motion error for this new frame will be:

$$e_{5}^{\prime }=\left(F_{6}-M\left(F_{5}\right)\right)+\left(N_{6}-M\left(N_{5}\right)\right).$$

Notice that for frames belonging to the same GOP, the components of the additive noise term $N_{i}-M\left(N_{i-1}\right)$ are correlated, thus, we can expect some noise cancellation. After the deletion of frame $P_{4}$, however, the two components of the additive noise term $\left(N_{6}-M\left(N_{5}\right)\right)$ are not correlated, leading to a relatively larger motion error compared to the others. This pattern can be learned by a deep neural network with sufficient training data samples.

### 3.2. Social Media Platform Identification Framework

In this section, we propose two learning methods to detect and classify different static and temporal recompression fingerprints left by social media platforms on shared videos exploiting a unified set of features. Through these learning methods, the goal is to evaluate the transferability of features between the image and video tasks and to show the hierarchical relation of these two tasks. In all the following sections, we construct our methods starting from the MISL network introduced by Bayar and Stamm [43] to train it with two different learning approaches. This network has proven successful in several multimedia forensics applications [1,14], so we decided to keep its architecture and optimize it for our setting. Because the MISL network was originally designed to work on greyscale images, we modified the initial constrained layer to work on RGB inputs, therefore, we doubled the number of kernels in the first convolutional layer from 3 to 6, to increase the expressive power of the network and match the more complex input the model is fed with. The network has 5 convolutional layers (called *constrained*, *conv1*, *conv2*, *conv3*, *conv4*) and three fully connected layers (called *fc1*, *fc2*, *fc3*). The *fc3* layer has a number of neurons corresponding to the number of output classes. The network is trained on RGB image patches for the image social media identification platform task, and on RGB I-frame and P-frame patches extracted from videos for the video source platform identification task. Differently from state-of-the-art methods reported in Section 2, we decided to use the constrained convolutional layer to automatically learn the best input transformation instead of feeding the network with DCT histograms or reference sensor pattern noise. Therefore, we train the network with RGB input patches extracted from video frames.

In the following sections, we use I and V to refer to the image task and video task respectively. Moreover, we use $X_{I}$ and $X_{V}$ to refer to the input image or video patches of the network and $Y_{I}$ and $Y_{V}$ to refer to the corresponding output classes.

#### 3.2.1. Method Based on Transfer Learning

In this section we propose transfer learning to transfer the static features learned by a base model on images to the video domain, so as to increase the performance of the same model on this new target task. Because we want the model to learn a certain fingerprint in both image and video sharing tasks, we adopt this technique to measure how features learned on one of the two tasks generalize to the other and study the hierarchical structure of features extracted at different layers of the network.

**Figure 3:**
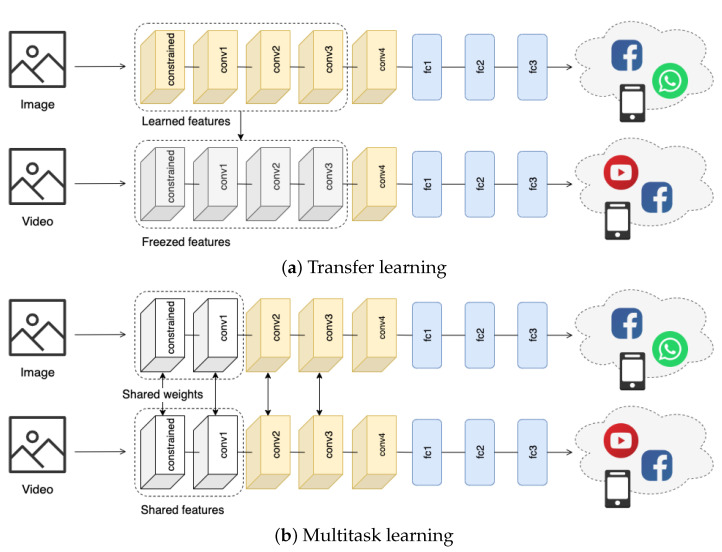
Learning approaches proposed in this paper: (**a**) Method based on transfer learning; (**b**) Method based on multitask learning. In the transfer-learning approach we initially train the model on the image task. Then we reuse the feature representations learned on images to train the model on the video source platform identification task. In multitask learning we share the weights of the *constrained* and *conv1* layers of two siamese networks while learning them on images and videos in parallel.

In this setting, we initially train the model with image RGB inputs $X_{I}$ to predict the platform of provenance $Y_{I}$ of these images. The network is initialized with a Xavier initializer [44] and trained on 256×256 input patches to predict the output classes with a cross-entropy loss function. As shown in Figure 3, we train this network on native single-compressed images (i.e., images that have not been shared on any platform) and images shared across social networks. Next, we perform feature transfer by freezing a number of layers from the image task and we retrain the remaining network layers on RGB patches $X_{V}$ extracted from video frames. We iterate this process starting from the lower *constrained* layer up to the higher *fc2* layer of the network. At each iteration, we freeze all the middle layers in between the constrained layer and the upper layer that we want to transfer. Figure 3 shows an example of this iterative feature-transfer approach. We initially train the model on the image task in a single-task learning fashion to predict the corresponding platforms of provenance. Then, we freeze all the convolutional layers from the *constrained* layer up to the *conv3* layer and retrain the remaining fully connected layers on the video task to predict the actual new social media platforms. In Section 4.3, we show that, according to the generic transfer learning behavior, low-level features generalize well across the two tasks, whereas deeper levels tend to learn more task-related representations. This information will be useful to understand how much the two tasks share with each other.

#### 3.2.2. Method Based on Multitask Learning

In multitask learning, we constrain some layers of two models to learn a unique set of parameters for different tasks. In this way, we encourage the shared layers of the network to learn a generalized representation that should help to produce more robust and flexible classifiers with respect to both static and temporal features. As we mentioned previously, the collection large datasets of shared multimedia contents is very hard because of several limitations (mostly related to privacy policies and API restrictions); this approach instead helps to train the network on smaller training datasets. Therefore, in this setting, we force the two networks to share a number of layers to learn more adaptable feature representations.

Figure 3 shows the multitask learning-based network used in this paper. In the figure, the two proposed networks share the weights from the *constrained* layer up to the *conv1* layer to learn a common feature extractor given input images $X_{I}$ and videos $X_{V}$. Next, the two networks independently learn to predict the correct output classes $Y_{I}$ and $Y_{V}$. Clearly, as suggested by the hierarchical dependencies of features maps extracted by different layers of the network highlighted by transfer learning, for these tasks it is not helpful to share all the layers from the *constrained* layer up to the *fc2* layer (see Section 4.4). Thus, to choose which layers to share, we use what we have learned with transfer learning by selecting the layers that extract the more general representations useful for both images and videos, that is, the constrained layer and *conv1* layer.

Because detecting forensics traces left by social media on videos is harder than learning such fingerprints on images [38], we train the multitask learner by taking this information into consideration and slow down the learning process on images. More precisely, we train the model measuring the cross entropy loss on each task and weighing the overall loss according to the following equation:

(1) $$L=\frac{1}{N}\left(w_{I}L_{I}+w_{V}L_{V}\right)$$

where $L_{I}$ and $L_{V}$ are the cross-entropy losses on images and videos respectively, *N* is the number of tasks (2 in our setting), and $w_{I}$ and $w_{V}$ are the weights assigned to each task. The weights can be experimentally adjusted on each task depending on the availability of training data and task complexity. In all our experiments, we fix $w_{I}=0.25$ and $w_{V}=1$ such as to reduce the loss on the image task and accelerate the improvements on videos. As for the method based on transfer learning, at each training iteration the weights and biases of the model are updated according to gradient descent $w^{\left(\ell \right)}=w^{\left(\ell \right)}-\alpha \frac{\partial L_{t}}{\partial w^{\left(\ell \right)}}$, where $L_{t}$ indicates the loss measured on task t∈{I,V} and $w^{\left(\ell \right)}$ represents the weights matrix at layer *ℓ*.

## 4. Experimental Evaluation

In this section, we experimentally evaluate the effectiveness of transfer learning and multitask learning with respect to a baseline single-task learning model fully trained on the target task. Specifically, (1) we measure the performance of two baseline single-task models trained on images and videos; (2) we evaluate the importance of hierarchical features with respect to images and videos, measuring the amount of information that the two tasks share at each level of depth through transfer learning; (3) we compare the results of the multitask-learning approach with those relative to transfer learning and single-task learning.

### 4.1. Dataset and Experimental Setting

We run our experiments on the VISION dataset [17]. The dataset includes 34,427 images and 1914 videos, both in the native format (original) and in their social media version (i.e., Facebook and WhatsApp for images, YouTube and WhatsApp for videos), captured by 35 portable devices of 11 major brands in many different settings. In our experiment, we split the dataset for training and validation with a proportion of 80% and 10%, respectively. Moreover, we use the remaining 10% of the dataset for testing purposes. All the results reported in this section refer to this set. This ensures the robustness of the model with respect to completely unseen data. Finally, we use the *ffprobe* (https://ffmpeg.org/ffprobe.html—accessed on 4 August 2021) analyzer to extract the I-frames and P-frames from all videos in the dataset and crop each frame and image into non-overlapping patches of size H×W, where H=W=256.

All experiments were carried on a Google Cloud Platform n1-standard-8 instance with 8 vCPUs, 30 GB of memory, and an NVIDIA Tesla K80 GPU. The models have been implemented using Pytorch (https://pytorch.org/—accessed on 4 August 2021) v.1.6. For the first two sets of experiments, we trained all the networks with the learning rate set to 1 × 10${}^{-4}$, a learning rate decay of 0.95 fixed at every epoch, weight decay set to 5 × 10${}^{-3}$, and AdamW optimizer. In our experiments, we trained the networks for 100 epochs with batches of size 64 and early stopping set to 10. Finally, to train the multitask model, we set a learning rate to 1 × 10${}^{-3}$, a learning rate decay of 0.99, and weight decay set to 1 × 10${}^{-2}$. The model was trained for 250 epochs with a batch size of 64. All models were initialized with a Xavier initializer [44].

### 4.2. Evaluation of Single-Task Learning

**Figure 4:**
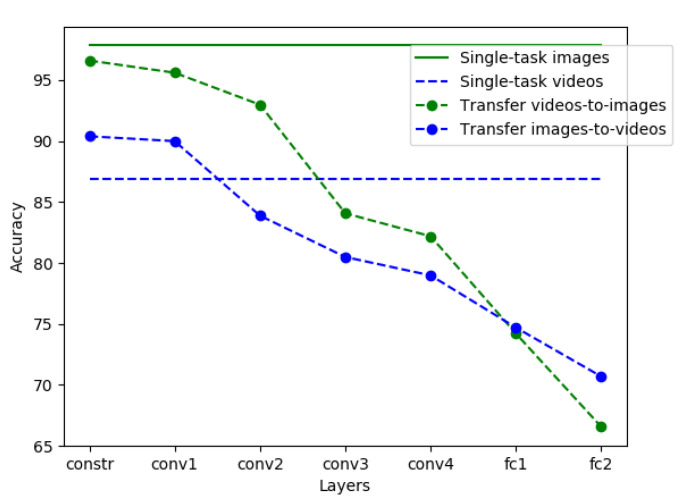
Comparison of baseline single-task learning, transfer-learning–based, and multitask-learning–based models accuracy on image (in green) and video (in blue) patches.

To measure the effect of transfer learning and multitask learning, we introduce a baseline model trained on each task. We trained the network on images and videos, measuring the model effectiveness on both tasks. In single task, we achieved an accuracy of 97.84% for RGB image patches and 86.85% for RGB video patches extracted from frames (see Figure 4). Interestingly, we did not observe substantial differences when training the model with both I-frame and P-frame video versus I-frame alone. However, we decided to keep both types of frames to help generalize the model by exposing it to as different cases as possible. Finally, to validate our choice to train the model on RGB patches without any preprocessing on the input, we compared the performance of our method with the Y-channel of the input after converting RGB to YUV, and we observed a decrease in accuracy of 1.41% for images and 4.2% for videos.

Table 1 and Table 2 report the confusion matrices of the single-task detectors on both tasks. Even though we do not apply any preprocessing operation to the input patches, the model achieves state-of-the-art performance comparable to the much more complex FusionNET [4] for the image task. Indeed, the FusionNET has 99.97%, 98.65%, and 99.81% patch-level accuracy on Facebook, WhatsApp, and native images, respectively, with an average difference of +1.89% with respect respect to our single-task model. For videos, our method suffers a drop in accuracy compared to the image task, but it still achieves results around 86.85%. Finally, we tested the overall accuracy of the model at image level and video level applying *majority voting* (i.e., the class that is voted by the majority of input patches is selected as the predicted class of the entire image or video), reaching 98.52% and 85.48%, respectively.

### 4.3. Evaluation of Transfer Learning

We performed a set of experiments to measure the robustness of methods based on transfer learning to images and videos. To perform the experiments, we froze some layers of the network with the learned parameters in one task and we retrained the remaining layers in the other task. To track the hierarchical dependencies of each task and measure the similarity of the two, we repeated this process for each level in the network from the *constrained* layer up to the *fc2* layer. As shown in Figure 4, the two tasks share low-level features, whereas deeper representations are mostly related to the target task with the accuracy varying from 66.56% to 96.60% for images and from 70.69% to 90.39% for videos at the patch level. On images (in green), the accuracy deteriorates as more layers are shared from the pretrained *constrained* layer up to the *fc2* layer. When knowledge is transferred from the image domain to the video domain (in blue), the network achieves 90.39% accuracy, gaining 3.54% accuracy with respect to the single-task model. This result confirms the intuition that lower-level features are shared between the two tasks, and that the *hierarchical* dependence between the two tasks can be used to train a deep-learning model on a small set of images or videos originating from social networks. In fact, the features extracted from the deeper levels turn out to be specific to the task being solved and therefore less generalizable, whereas the features extracted from the first levels of the network are more generic and, therefore, can be shared between the two tasks. The accuracy increases when measuring the performance at the image and at the video level. Specifically, the accuracy on images varies from 80.15% to 97.87%, with maximum accuracy up to 98.37% obtained by transferring video features up to the *conv2* layer. Finally, when transferring from images to video, we can observe an increase in accuracy from 85.48% to 92.61% on the video classifier, but the same does not happen for the transfer from video to images. This result can probably be explained by considering the videos as a more specific case and then thinking of this task as a subset of the corresponding task on images, thus suggesting an *asymmetry* between the two tasks.

### 4.4. Evaluation of Multitask Learning

**Figure 5:**
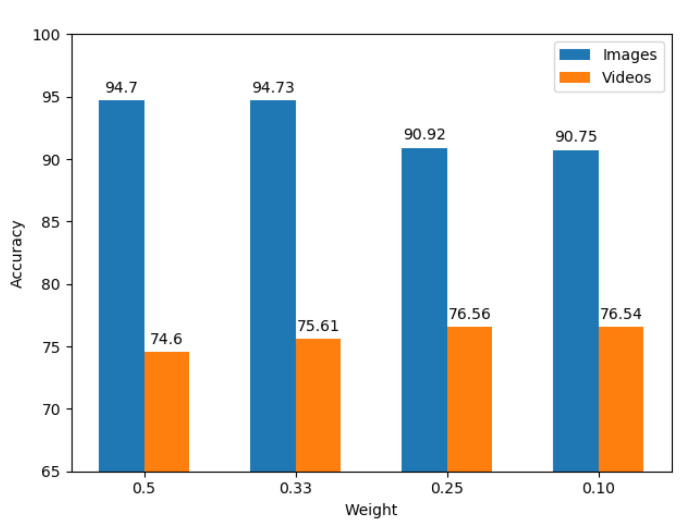
Test accuracy of the multitask learner on images and video I-frames obtained by fixing $w_{V}=1$ and varying the images weight $w_{I}$ according to Equation (1).

With this last experiment, we measured the performance of the proposed multitask learner. Specifically, we chose to train two networks on both tasks, by forcing them to share weights between the first two convolutional layers, namely the *constrained* and *conv1* layers. Because of the different complexity of the two tasks highlighted by transfer learning, it is not useful to share all the layers between the two networks and it becomes necessary to balance the learning speed on images with compared to the videos. Therefore, we initially run several experiments with variable weighted loss according to Equation (1). To speed up the training, in this exploratory phase we chose to train the networks on images and I-frames only for the videos. We report the results of this experiment in Figure 5. We have varied the images weight $w_{I}$ from 0.5 down to 0.1. Then, we chose $w_{I}=0.25$ so as to maximize the accuracy of the multitask learner on the video task and we retrained the multitask-learning-based model sharing the *constrained* and *conv1* layers between the two tasks. In this configuration, the multitask-learning-based model achieved 85.91% accuracy on images and 81.70% accuracy on videos. Finally, we tested the overall accuracy of the model at the image and the video level, reaching 92.08% and 91.55% accuracy on the images and the videos respectively. In this setting the model reaches an accuracy comparable to the single-task learner for the video task.

To evaluate the performance of our method, we compared it with the state-of-the-art two-stream network introduced by Amerini et al. [39]. To compare the performance of the transfer-learning and multitask-learning–based methods with that of Amerini et al. [39], we retrained the model of that work in this new setting. Table 3 shows the results of this comparison. Splitting the dataset at video level instead of frame level, the method from Amerini et al. [39] records a drop in accuracy of 15.47% compared to the configuration used in the original paper.

## 5. Discussion

While the method based on transfer learning achieves a higher overall accuracy than the one based on multitask learning, we investigated the different performance of these two approaches. To analyze and compare the results of the two methods, we kept the best configuration of the multitask learning-based model and examined the results of the transfer learning-based model when transferring features from the *constrained* and *conv1* layers as for the multitask network. Table 4 shows the confusion matrices of these two methods on videos.

First, the transfer-learning model is able to achieve better results than the baseline model on YouTube and native videos (see Table 2 and Table 4a). However, the WhatsApp class gets more easily confused with the other classes. Second, the multitask learner (Table 4b) tends to learn features representations that are more equally separated, with accuracy on all classes that oscillates between 79.25% and 83.68%, making the multitask learner less biased and more robust across all the classes. Moreover, thanks to this property, the multitask approach introduces an improvement in classification performance on WhatsApp compared to transfer learning (+10.74%, see Table 4) and the baseline model (+7.89%, see Table 2 and Table 4b). Because WhatsApp is the only class shared by the image and video sets, it might suggest that training a model in a multitask setting on images and videos from the same social media platform could be even more beneficial. To evaluate this intuition we tested the model on WhatsApp with native images and videos, achieving encouraging results. The multitask-learning model achieves higher accuracy than transfer learning and single-task learning, again obtaining more stable accuracy across all classes. Most likely, images and videos shared through the same platform use very similar compression algorithms, favoring the learning of the alterations introduced when the content is recompressed when uploaded to the platform. Table 5b,c show the results of this experiment. However, because of the lack of publicly available datasets containing both images and videos we are not able to verify whether this is the case with more classes and leave this issue open for future research.

## 6. Conclusions

In this paper, we propose two methods to identify the platform of origin of videos shared on different social networks through the use of joint features from images. Moreover, we show that images and videos share common forensic traces and a mixed approach may be beneficial in some cases where data are not enough to train a single-task model. By applying a transfer-learning–based method on both tasks, we experimentally showed that: (1) As expected, low-level features generalize well across images and videos, whereas deeper-feature mappings are more related to the target task, therefore suggesting that a common feature hierarchy exists between the two tasks; (2) image features can be successfully used to identify the social media platform in which videos have been uploaded, helping to improve performance over single task learning. Finally, we showed the promising effectiveness of a multitask-learning approach compared to single-task learning. In this way, the model can learn from images and videos simultaneously, learning more generic and robust features across all classes. These findings suggest that learning from multiple media could help to overcome the hurdle of training low-data models, by taking advantage of the similarity of different forensic tasks, usually treated separately.

Future work could be aimed at gathering a larger training dataset for social-media–platform identification of multimedia content and at studying the case of multiple sharing considering both images and videos. Moreover, a limitation of our method is that it appears susceptible to false positive classifications, leaving room for improvement.

## Figures and Tables

**Table 1:** Confusion matrix of the baseline single-task model on patches extracted from images. FBH and FBL represent high-quality and low-quality patches from Facebook. WA and NAT represent WhatsApp and native image patches respectively.

	FB	WA	NAT
FB	**98.78%**	0.05%	1.17%
WA	0.23%	**98.37%**	1.40%
NAT	1.56%	1.31%	**97.13%**

**Table 2:** Confusion matrix of the baseline single-task model on patches extracted from video frames. YT, WA and NAT represent YouTube, WhatsApp and native video patches respectively.

	YT	WA	NAT
YT	**85.28%**	8.36%	6.45%
WA	11.56%	**72.35%**	16.09%
NAT	2.85%	11.15%	**86.00%**

**Table 3:** Comparison of video patch classification accuracy of our transfer-learning and multitask-learning methods with the one of Amerini et al. [39] on the VISION dataset.

Method	Accuracy
[39]	80.04%
TL (ours)	**90.39%**
MT (ours)	81.70%

**Table 4:** Confusion matrices on video patches of the transfer-learning (a) and multitask learning (b) models sharing the *constrained* and *conv1* layers.

(a) Transfer Learning			
	YT	WA	NAT
YT	**91.24%**	1.08%	7.66%
WA	13.33%	**69.50%**	17.15%
NAT	6.05%	1.49%	**92.45%**
**(b) Multitask Learning**			
	YT	WA	NAT
YT	**83.68%**	6.19%	10.04%
WA	10.04%	**80.24%**	9.72%
NAT	10.58%	10.17%	**79.25%**

**Table 5:** Confusion matrices on video patches of the transfer-learning (a) and multitask learning (b) models sharing the *constrained* and *conv1* layers.

(a) Single-Task Learning		
	WA	NAT
WA	**60.12%**	39.88%
NAT	28.07%	**71.93%**
**(b) Transfer Learning**		
	WA	NAT
WA	**63.08%**	36.92%
NAT	23.69%	**76.30%**
**(c) Multitask Learning**		
	WA	NAT
WA	**71.48%**	28.52%
NAT	26.16%	**73.84%**

## Data Availability

No new data were created in this study.
